# Dietary restriction delays aging, but not neuronal dysfunction, in *Drosophila* models of Alzheimer's disease

**DOI:** 10.1016/j.neurobiolaging.2009.10.015

**Published:** 2011-11

**Authors:** F. Kerr, H. Augustin, M.D.W. Piper, C. Gandy, M.J. Allen, S. Lovestone, L. Partridge

**Affiliations:** a Institute of Healthy Ageing, and GEE (Genetics, Evolution and Environment), University College London, Gower Street, London WC1E 6BT, UK; b School of Biosciences, University of Kent, Canterbury CT2 7NJ, UK; c MRC Centre for Neurodegeneration Research, Institute of Psychiatry, King's College London, London, SE5 8AF, UK; d Max Planck Institute for Biology of Ageing, Gleueler Straße 50a, Köln, Germany

**Keywords:** DR, dietary restriction, AD, Alzheimer's disease, Aβ, amyloid-beta, NFT, neurofibrillary tangle, APP, amyloid precursor protein, FAD, familial Alzheimer's disease, LOAD, late-onset Alzheimer's disease, IIS, insulin/IGF-like signalling, IF, intermittent fasting, AL, *ad libitum*, SY, sugar-yeast, WT, wild-type, UAS, upstream activator sequence, PI, performance index, DT, decline time, GFS, giant fibre system, GF, giant fibre, TTM, tergotrochanteral muscle, DLM, dorsal longitudinal flight muscle, Alzheimer's disease, Aging, Dietary restriction, Neuronal function, *Drosophila*

## Abstract

Dietary restriction (DR) extends lifespan in diverse organisms and, in animal and cellular models, can delay a range of aging-related diseases including Alzheimer's disease (AD). A better understanding of the mechanisms mediating these interactions, however, may reveal novel pathways involved in AD pathogenesis, and potential targets for disease-modifying treatments and biomarkers for disease progression. *Drosophila* models of AD have recently been developed and, due to their short lifespan and susceptibility to genetic manipulation, we have used the fly to investigate the molecular connections among diet, aging and AD pathology. DR extended lifespan in both Arctic mutant Aβ42 and WT 4R tau over-expressing flies, but the underlying molecular pathology was not altered and neuronal dysfunction was not prevented by dietary manipulation. Our data suggest that DR may alter aging through generalised mechanisms independent of the specific pathways underlying AD pathogenesis in the fly, and hence that lifespan-extending manipulations may have varying effects on aging and functional declines in aging-related diseases. Alternatively, our analysis of the specific effects of DR on neuronal toxicity downstream of Aβ and tau pathologies with negative results may simply confirm that the neuro-protective effects of DR are upstream of the initiating events involved in the pathogenesis of AD.

## 1 Introduction

Alzheimer's disease (AD) is a highly prevalent and devastating aging-related condition. Clinically, AD patients display progressive cognitive decline, including deficits in memory, function and behaviour. AD is characterised pathologically by the presence of both extracellular amyloid-beta (Aβ) plaques and intraneuronal neurofibrillary tangles (NFTs), composed of the microtubule-associated protein tau, which occur mainly in regions of the brain involved in learning and memory. AD aetiology is multifactorial, with both genetic and environmental factors implicated in its pathogenesis. Familial forms of the disease occur at an early age and are caused by autosomal dominant mutations in Presenilins 1 and 2 or the amyloid precursor protein (APP). More commonly, however, AD occurs late in life (late-onset AD; LOAD) and the aetiological processes underlying this form of the disease are not well understood. Since aging is the most common risk factor for AD, it seems probable that molecular mechanisms underlying the aging process also play a role in the initiation of AD pathogenesis. Some recent evidence from the study of model organisms suggests that this is indeed the case. For instance, attenuation of insulin-like signalling (IIS) and dietary restriction both extend lifespan and protect against Aβ and, in the case of DR, against other types of proteotoxicity in genetic models of disease in the nematode worm *Caenorhabditis elegans* (Cohen et al., 2006; Steinkraus et al., 2008). Investigation of the molecular mechanisms by which ameliorating the aging process can protect against AD pathology should highlight important pathways involved in the initiation of sporadic AD pathogenesis, and hence lead to the discovery of novel targets for disease-modifying therapies and biomarkers for the progression of the disease.

Dietary restriction (DR), a reduction in food intake that is not accompanied by malnutrition, extends lifespan in many organisms including the yeast *Saccharomyces cerevisiae* (Lin et al., 2002), *C. elegans* (Klass, 1977; Houthoofd et al., 2003), the fruit fly *Drosophila melanogaster* (Chippindale et al., 1993; Chapman and Partridge, 1996; Mair et al., 2003) and rodents (McCay et al., 1935; Masoro, 2005). DR has also been shown to delay several aging-related functional declines and disease processes (Bishop and Guarente, 2007; Mair and Dillin, 2008). Many studies have reported beneficial effects of DR on aging-associated cognitive defects in rodents (Pitsikas et al., 1990; Markowska and Savonenko, 2002; Adams et al., 2008), although others have also reported no positive effect (Bellush et al., 1996; Markowska, 1999; Fitting et al., 2008) and the mechanism of protection, where it occurs, remains to be established. Diets high in fat and calories have been implicated as an important risk factor for AD (Luchsinger et al., 2002; Luchsinger and Mayeux, 2004). Moreover, dietary restriction has been shown to modify the molecular neuropathology of the disease in animal models. DR confers protection against excitotoxic damage in mouse neurons expressing a familial AD mutation in presenilin-1 (Zhu et al., 1999), reduces Aβ deposition (Patel et al., 2005; Wang et al., 2005; Halagappa et al., 2007), reduces tau hyperphosphorylation (Halagappa et al., 2007; Wu et al., 2008) and improves cognitive dysfunction in mouse models of AD (Halagappa et al., 2007). Importantly, different methods of DR have been shown to ameliorate cognitive decline in aged mice displaying both Aβ and tau pathologies (Halagappa et al., 2007), but varying mechanisms have been proposed. DR can be implemented in rodents by restricting the amount of food available for consumption to a fixed percentage of the amount of standard laboratory diet eaten by the *ad libitum* fed controls (Piper and Bartke, 2008), and is thought to assert its neuro-protective effects upstream of Aβ and tau pathologies by preventing their abnormal production (Halagappa et al., 2007). Alternatively, intermittent fasting (IF), where animals have no access to food for 24 h and *ad libitum* (AL) access to food during the next 24 h (Piper and Bartke, 2008), is thought to act downstream of AD pathogenesis (Halagappa et al., 2007). The precise mechanisms mediating these effects, however, are unclear and whether there is a molecular connection between the effects of DR on aging and AD-associated declines in cognition remains to be established.

Most of our current understandings of the molecular determinants of aging in response to DR have come from the study of invertebrate model organisms such as yeast, worms and flies (Bishop and Guarente, 2007; Mair and Dillin, 2008). Conserved regulators of DR longevity remain elusive, but insulin-like signalling (IIS), target of rapamycin (TOR), AMP-dependent protein kinase (AMPK) and sirtuin pathways are candidate mechanisms for the regulation of DR-dependent lifespan extension in multiple species (Bishop and Guarente, 2007; Mair and Dillin, 2008). Moreover, several studies have implicated components of the IIS and sirtuin pathways in mediating the neuro-protective effects of DR on aging-related cognitive decline in rodents (Martin et al., 2006; Gillette-Guyonnet and Vellas, 2008).

*D. melanogaster* is a useful model organism in which to further study the complex interactions between DR, aging and AD due to its short lifespan, well-differentiated brain and susceptibility to genetic manipulation. *Drosophila* models of Alzheimer's disease have recently been developed and recapitulate separately many features of Aβ and tau pathologies, including formation of amyloid plaques and neurofibrillary tangles, neuronal death, behavioural impairment and shortened lifespan (Wittmann et al., 2001; Crowther et al., 2005). Dietary restriction protocols are well established in flies, most commonly implemented by diluting the concentration of nutrients in the fly food medium (Chapman and Partridge, 1996; Bass et al., 2007; Wong et al., 2008, 2009), a method analogous to food restriction regimes used in rodents. We have, therefore, examined the effects of DR on lifespan and on biochemical and neuronal dysfunction phenotypes in flies over-expressing human Aβ42 peptides or 4R tau protein in the nervous system, with a view to dissecting the molecular mechanisms connecting DR, aging and AD.

Our study firstly extends the characterisation of fly AD models beyond previously published studies using electrophysiological methods to directly measure neuronal activity in Aβ42 and tau over-expressing flies. We propose that this additional measurement of synaptic activity, in combination with previously established behavioural phenotypes, will provide a better output response for the analysis of early events involved in the neuro-pathogenesis of AD in the fly. Our findings also show that although DR extends lifespan, it has no impact on Aβ or tau pathologies in the nervous system and it does not protect against neuronal dysfunction in these *Drosophila* models of AD. DR longevity may dissociate from the specific pathways underlying AD pathogenesis that we examined, and supports the view that lifespan and aging-related pathology can be affected by different mechanisms. On the other hand, because our *Drosophila* study specifically examines the effects of DR on events downstream of Aβ and tau accumulation, our findings may concur with those in previous reports for mice (Halagappa et al., 2007) that any protective effects of DR act upstream of the initiating events in the pathogenesis of Alzheimer's disease.

## 2 Materials and methods

### 2.1 Fly stocks and maintenance

All fly stocks were maintained and experiments conducted at 25 °C on a 12:12-h light:dark cycle at constant humidity using standard sugar-yeast medium. Neuron-specific expression of Arctic mutant Aβ42 peptide or WT 4R tau proteins was achieved with the GAL4-UAS system [GAL4-dependant upstream activator sequence (Brand and Perrimon, 1993)]. The Aβ42 and tau UAS lines were obtained as generous gifts: UAS-ArcAβ42 (Dr D. Crowther, Cambridge, UK) and UAS-WT 4R tau (Dr E. Skoulakis, Vari, Greece; originally sourced from Prof. M. Feany, Harvard Medical School, USA). Elav GAL4^C155^ stocks were obtained from the Bloomington stock centre. All transgenes were backcrossed six times into the w^1118^ genetic background. Female flies carrying UAS-ArcAβ42 or UAS-WT 4R tau constructs were crossed to male flies with the pan-neuronal GAL4 driver elavGAL4^C155^ on the X chromosome. Female F1 progeny carried both UAS and GAL4 constructs and were used for subsequent analyses.

### 2.2 DR by yeast dilution series

Flies were raised at a standard density of 50 larvae per vial on 1.0 sugar-yeast (SY) medium (15 g l^−1^ agar, 50 g l^−1^ sugar, 100 g l^−1^ autolysed yeast, 100 g ^−1^ nipagin and 3 ml l^−1^ propionic acid). Two days after eclosion females were separated from males and transferred to experimental vials, 10 flies per vial. Dietary restriction (DR) was implemented with a constant sugar concentration (50 g l^−1^) and a variable yeast concentration (× Y; 0.1/10 g l^−1^, 0.5/50 g l^−1^, 1.0/100 g l^−1^, 1.5/150 g ^−1^ and 2.0/200 g ^−1^). The yeast source was that referred to as SY Brewer's in Bass et al. (2007).

### 2.3 Lifespan analyses

Deaths were scored almost every day and flies were transferred to fresh food three times a week. Statistical comparisons of survival between genotypes were made using log-rank tests at each food concentration, and within genotypes between food concentrations to determine the food dilution that maximised lifespan.

### 2.4 Negative geotaxis assays

Negative geotaxis was analysed using 1.0 (DR) versus 2.0 (fully fed) Y medium at various fly ages (1, 5, 10, 15, 25, 30 and 40 days old). Assays were performed according to previously published methods (Rival et al., 2004). Briefly, 15 adult flies were anaesthetised and placed in a vertical column (25 cm long, 1.5 cm diameter) with a conic bottom end and allowed to recover from CO_2_ exposure for 30 min. For assays, flies were tapped to the conic end of the column, then their climbing to the top of the column analysed. Flies reaching the top of the column or remaining at the bottom after a 45 s period were counted. Three trials were performed at 1 min intervals for each experiment. The mean number of flies at the top (*n*_top_), the mean number of flies at the bottom (*n*_bottom_) and the total number of flies assessed (*n*_tot_) were recorded.

Climbing ability was then analysed using several functional descriptors as previously described (Martin et al., 2006): negative geotaxis data were calculated as a performance index (PI) defined as 1/2(*n*_tot_ + *n*_top_ − *n*_bottom_)/*n*_tot_. DT50 (time for function to decline to 50% of peak value) constant was extrapolated from second-order polynomial curve fits of the negative geotaxis data, and the total climbing function across ages was calculated as the area under the negative geotaxis curve for each group, using Prism 5.0 software (GraphPad software, San Diego, CA, USA).

### 2.5 Electrophysiology

Recordings from the giant fibre system (GFS) of adult flies were made as described in Allen et al. (1999); a method based on those described by Tanouye and Wyman (1980) and Gorcyzca and Hall (1984). Flies were anaesthetized by cooling on ice and secured in wax, ventral side down, with the wings held outwards. A tungsten earth wire (ground electrode) was placed into the abdomen; tungsten electrodes were pushed through the eyes and into the brain to deliver a 40 V pulse for 0.03 ms using a Grass S48 stimulator. Recordings were made from the tergotrochanteral muscle (TTM) and contralateral dorsal longitudinal muscle (DLM) using glass microelectrodes (resistance: 40–60 MΩ). The electrodes were filled with 3 M KCl and placed into the muscles through the cuticle. Responses were amplified using Getting 5A amplifiers (Getting Instruments, USA) and the data digitized using an analogue-digital Digidata 1320 and Axoscope 9.0 software (Axon Instruments, USA). For response latency recordings, at least 5 single stimuli were given with 5 s rest periods between each stimulus; trains of 10 stimuli, at either 100, 200 or 250 Hz, were given with a 5 s rest interval between each train.

### 2.6 Quantification of total, soluble and aggregated Aβ42

To extract total Aβ42, five fly heads were homogenised in 50 μl GnHCl extraction buffer (5 M Guanidinium HCl, 50 mM Hepes pH 7.3, protease inhibitor cocktail (Sigma, P8340) and 5 mM EDTA), centrifuged at 21,000 × *g* for 5 min at 4 °C, and cleared supernatant retained as the total fly Aβ42 sample. Alternatively, soluble and insoluble pools of Aβ42 were extracted using a protocol adapted from previously published methods (Burns et al., 2003): 200 fly heads were homogenised in 200 μl tissue homogenisation buffer (250 mM sucrose, 20 mM Tris base, 1 mM EDTA, 1 mM EGTA, protease inhibitor cocktail (Sigma)) then mixed further with 200 μl DEA buffer (0.4% DEA, 100 mM NaCl and protease inhibitor cocktail). Samples were centrifuged at 135,000 × *g* for 1 h at 4 °C (Beckman Optima™ Max centrifuge, TLA120.1 rotor), and supernatant retained as the cytosolic, soluble Aβ42 fraction. Pellets were further resuspended in 400 μl ice-cold formic acid (70%), and sonicated for 2× 30 s on ice. Samples were re-centrifuged at 135,000 × *g* for 1 h at 4 °C, then 210 μl of supernatant diluted with 4 ml FA neutralisation buffer (1 M Tris base, 0.5 M Na_2_HPO_4_, 0.05% NaN_3_) and retained as the aggregated, formic acid-extractable Aβ42 fraction.

Total, soluble or aggregated Aβ42 content was measured in Arctic mutant Aβ42 flies and controls using the hAmyloid-β42 ELISA kit (HS) (The Genetics Company, Switzerland). Total and soluble Aβ42 samples were diluted 1:10, and aggregated Aβ42 samples diluted 1:5 in sample/standard dilution buffer and the ELISA performed according to the manufacturers’ instructions. Protein extracts were quantified using the Bradford protein assay (Bio-Rad protein assay reagent; Bio-Rad laboratories Ltd. (UK)) and the amount of Aβ42 in each sample expressed as a ratio of the total protein content (pmol/g total protein).

### 2.7 Analysis of tau phosphorylation by western blotting

Tau phosphorylation was analysed in WT 4Rtau over-expressing flies and controls by western blotting. 20 *Drosophila* heads were homogenised in 2× Laemmli sample buffer containing β-mercaptoethanol and boiled for 5 min. Proteins were separated on 10% sodium dodecyl sulfate–polyacrylamide (SDS–PAGE) gels at 150 V for 1 h. Gels were then transferred to nitrocellulose membranes, incubated in a blocking solution containing 5% milk proteins in TBST for 1 h at room temperature, then probed with primary antibodies overnight at 4 °C. The primary antibodies used were: anti-tau polyclonal antibody (DAKO, UK) is phosphorylation independent and recognises all tau isoforms, monoclonal antibody PHF-1 recognises tau phosphorylated at epitope Ser396/404 and was kindly provided by Dr P. Davies (Albert Einstein College of Medicine, NY, USA), monoclonal antibody AT8 (Innogenetics) recognises tau phosphorylated at the Ser199/202 epitope, polyclonal antibodies pS422 and actin (Abcam, UK) recognise the Ser422 phospho-epitope of tau and total actin proteins respectively. Anti-horseradish peroxidase (HRP)-conjugated secondary antibodies were used and blots were developed using the enhanced chemiluminescence method (ECL; Amersham Biosciences). The relative tau immunoreactivity for each antibody was analysed using Image J software (National Institutes of Health). Absolute intensity of phosphorylated tau was normalised to the total tau protein present in each sample (relative intensity).

### 2.8 Statistical analyses

Data are presented as means ± SEM obtained in at least three independent experiments. Log-rank tests, analysis of variances and Tukey's HSD (honestly significant difference) post-hoc analyses were performed using JMP (version 7.0) software (SAS Institute, Cary, NC, USA).

## 3 Results

### 3.1 Dietary restriction extends lifespan in *Drosophila* models of Alzheimer's disease

**Fig. 1:**
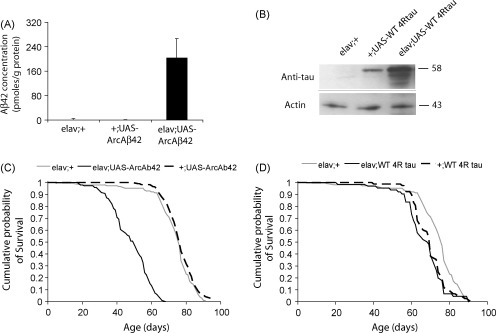
Biochemical confirmation of protein over-expression in Arctic Aβ42 and WT 4Rtau female flies. Flies were reared and maintained on 1.0 SY medium, then protein content quantified at 15 days old. (A) Detection of Aβ42 peptides in w^1118^elav/+;UAS-ArcAβ42/+ and control (w^1118^elav/+, w^1118^w^1118^;UAS-ArcAβ42/+) flies by sandwich ELISA (the Genetics Company; see Section 2). Data are presented as means ± SEM. *P* < 0.05 comparing w^1118^w^1118^elav/+;UAS-ArcAβ42/+ to both controls (Tukey's HSD). (B) Analysis of WT human 4R tau in w^1118^elav/+;UAS-WT 4Rtau/+ and control (w^1118^elav/+, w^1118^;UAS-WT 4Rtau/+) flies by western blotting using a non-phosphorylation-dependent anti-tau antibody (Dako; see Section 2). Lifespans were determined on standard 1.0 SY medium for flies over-expressing (C) Arctic Aβ42 peptides or (D) WT 4R tau compared to their GAL4 and UAS controls, and survival curves are depicted.

Over-expression of human Arctic Aβ42 and wild-type tau proteins was confirmed in flies heterozygous for both elav GAL4 and the UAS-ArcAβ42 (w^1118^elav/+;UAS-ArcAβ42/+) or UAS-WT4Rtau (w^1118^elav/+;UAS-WT4Rtau/+) transgenes respectively, compared to control flies expressing elav GAL4 (w^1118^elav/+) or UAS-ArcAβ42 (w^1118^;UAS-ArcAβ42/+) or UAS-WT4Rtau (w^1118^;UAS-WT4Rtau/+) alone (Fig. 1A and B). Consistent with previously published studies (Crowther et al., 2005), Arctic Aβ42 over-expressing flies showed substantially shortened lifespan compared to the elav GAL4 and UAS-ArcAβ42 transgene controls (Fig. 1C). In contrast, WT 4R tau over-expressing flies did not differ in lifespan from the UAS-WT 4R tau transgene control (Fig. 1D), although survival of both of these lines was substantially reduced in comparison to the elav GAL4 driver line. Western blotting of whole fly heads revealed that the UAS-WT 4R tau transgene was leaky (Fig. 1B). Leaky expression of tau in tissues other than neurons may therefore account for the reduction in lifespan of both the UAS-control line and the tau over-expressing flies compared to the GAL4 line alone. Alternatively, the level of leaky expression in the nervous system itself may have been sufficient to reduce lifespan in the UAS-control line.

**Fig. 2:**
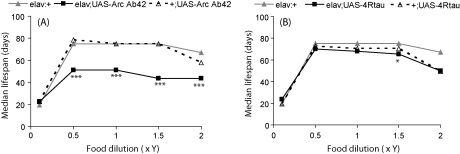
Median lifespans of flies over-expressing Arctic Aβ42 peptides or WT human 4R tau across a yeast dilution series on SY food. Lifespans of (A) w^1118^elav/+;UAS-ArcAβ42/+ or (B) w^1118^elav/+;UAS-4Rtau/+ and their control (w^1118^elav/+, w^1118^;+/UAS-ArcAβ42, w^1118^;+/UAS-4Rtau) females were performed across a yeast dilution series (0.5S, variable Y; see Section 2). Flies were reared at a standard density on 1.0 SY, and at day 3 mated females were distributed onto the appropriate 0.1, 0.5, 1.0, 1.5, 2.0× Y medium. Lifespans were determined (as described in Section 2) and median lifespan values for each group across the yeast dilution series are plotted. *P < 0.01, ***P < 0.000001 comparing survival of w^1118^elav/+;UAS-ArcAβ42/+ or w^1118^elav/+;UAS-4Rtau/+ to that of both control groups (log-rank tests). w^1118^elav/+ flies were the same for both Aβ42 and tau experiments which were run in parallel.

DR can be achieved in flies by dilution of the nutrients in their food medium (Chapman and Partridge, 1996). As food concentration declines from a maximum, lifespan first increases to a maximum in response to DR, before declining due to starvation at lower food concentrations. However, the particular diet that maximises lifespan can vary across different *Drosophila* strains, and so was empirically determined for each of the lines used in the current study using a range of food concentrations in which the yeast component was diluted to 0.1, 0.5, 1.0, 1.5 and 2.0× yeast (Y) compared to the standard SY medium (Fig. 2A and B). Although the DR peaks were broad in some cases (Table 1; see footnotes †, ‡), dietary responses for all genotypes were: starvation at 0.1 Y, DR at 0.5 to 1.5 Y and fully fed at 2.0 Y (Fig. 2 A and B, and Table 1). Arctic Aβ42 and tau reduced lifespan phenotypes were not apparent on 0.1 Y medium (Fig. 2A and B) compared to the elav GAL4 control line, presumably because all genotypes were starved, resulting in death prior to the onset of mortality caused by the over-expression of Aβ42 or tau proteins. Over-expression of Arctic Aβ42, however, did shorten lifespan compared to UAS and GAL4 controls on all other food concentrations studied (Fig. 2A). A reduction in food concentration from the fully fed condition increased the lifespan of Arctic Aβ42 flies, but this was insufficient to rescue survival back to the level of control flies, and the lifespan curves for the experimental and control genotypes remained roughly parallel throughout the DR food dilution range (Fig. 2A). DR thus neither rescued nor ameliorated the reduction in lifespan caused by Aβ pathology in this *Drosophila* model. On the other hand, despite the lack of a neuronal-specific effect of tau on lifespan at all yeast concentrations, nutritional dilution did improve the survival of both tau transgenic lines (w^1118^elav/+;UAS-WT4Rtau/+, w^1118^;UAS-WT4Rtau/+), and completely rescued the effect of leaky tau expression on reduced longevity compared to elav GAL4 controls at the 0.5 Y concentration (Fig. 2B and Table 1). Hence, DR does appear to protect against the toxic effect of tau on lifespan but the effect could be mediated in tissues other than the nervous system, since the rescue is similar for w^1118^elav/+;UAS-WT4Rtau/+ and w^1118^;UAS-WT4Rtau/+ flies.

Overall these findings confirm a lifespan-extending effect of DR under our experimental conditions in these *Drosophila* models of AD, but point to mechanisms that may be uncoupled from the neuron-specific defects.

### 3.2 Dietary manipulation does not alter the abnormal accumulation of Aβ42 peptides or tau in *Drosophila*

**Fig. 3:**
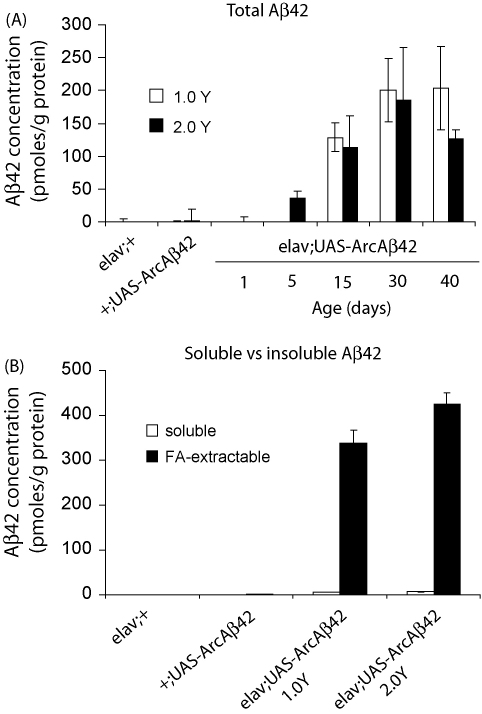
Biochemical analysis of fully fed vs DR food effects on Aβ42 levels and aggregation in flies over-expressing Arctic mutant Aβ42. (A) Dietary manipulation effects on Aβ42 accumulation in w^1118^elav/+;UAS-ArcAβ42/+ female flies across age. Flies were aged on the appropriate 1.0 or 2.0 Y medium and total Aβ42 levels measured by ELISA at the indicated timepoints. Data are presented as the mean ± SEM of the Aβ42 concentration in pmol/g total protein. Dietary effects on total Aβ42 levels across age in w^1118^elav/+;UAS-ArcAβ42/+ flies were analysed using a two-way ANOVA (*n* = 3). *P* = 0.267 comparing w^1118^elav/+;UAS-ArcAβ42/+ on 1.0 Y to that of flies on 2.0 Y, *P* = 0.0001 comparing Aβ42 levels across age. Tukey's HSD analysis revealed significant differences in Aβ42 levels in flies aged for 30 or 40 days compared to those at 1, 5 and 15 days old (*P* < 0.05). (B) Dietary manipulation effects on Aβ42 aggregation in w^1118^elav/+;UAS-ArcAβ42/+ female flies. Soluble and aggregated (formic acid-extractable) Aβ42 levels were measured by ELISA following 15 days treatment on the DR regime. Data are presented as means ± SEM. Dietary effects on each pool of Aβ42 in w^1118^elav/+;UAS-ArcAβ42/+ flies were analysed by two-way ANOVA (*n* = 3; *P* < 0.0001, comparing soluble vs aggregated Aβ42; *P* = 0.057, comparing Aβ42 on 1.0 vs 2.0 Y medium).

**Fig. 4:**
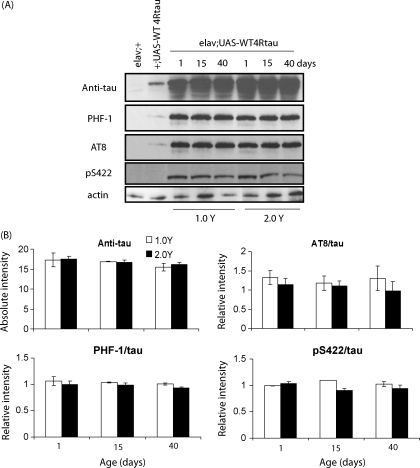
Analysis of fully fed vs DR food effects on tau levels and phosphorylation in flies over-expressing WT human tau. (A) Tau expression and phosphorylation levels were measured by western blotting in control flies (w^1118^elav/+, w^1118^;UAS-4Rtau/+), and at the indicated timepoints in w^1118^elav/+;UAS-4Rtau/+ flies treated on 1.0 vs 2.0 Y medium. Primary antibodies were as follows: Anti-tau (total tau; Dako, UK), PHF-1 (phospho-Ser396/404 tau), AT8 (phospho-Ser199/202 tau), pS422 (phospho-Ser422 tau) and anti-actin. (B) Phospho-tau levels, in w^1118^elav/+;UAS-4Rtau/+ flies, were normalised to total tau protein for each sample, and are expressed as average relative intensities ± SEM. Dietary manipulation did not alter the level or pattern of tau phosphorylation across age at Ser396/404 (*P* = 0.412), Ser199/202 (*P* = 0.838) or Ser422 (*P* = 0.677) epitopes (two-way ANOVA).

Since lifespan peaked between 0.5 and 1.5 Y and all genotypes showed reduced lifespan on 2.0 Y medium, further analyses of the effects of diet on AD pathology were performed using 1.0 Y as ‘DR’ food and 2.0 Y as ‘fully fed’ food. As a measure of early changes in the molecular pathology of AD in response to diet, Aβ42 accumulation and tau phosphorylation levels were assessed in Arctic Aβ42 and WT 4R tau over-expressing flies, respectively, across age on DR versus fully fed dietary regimen (Figs. 3 and 4).

w^1118^elav/+;UAS-ArcAβ42/+ flies did not express detectable levels of total (soluble and insoluble) Aβ42 peptides upon eclosion, but Aβ42 accumulated to detectable levels at 15 days old peaked at 30 days old (Fig. 3A). Dietary manipulation did not affect either the time-course or expression of Aβ42 peptide (Fig. 3A; grey vs black bars). Aβ42 transcript levels were also unaltered by changes in diet (Fig. S1A). Soluble and aggregated Aβ42 levels were further assessed in 15 days old w^1118^elav/+;UAS-ArcAβ42/+ flies. At this time-point most Aβ42 was distributed in the insoluble, formic acid-extractable pool with very little soluble Aβ42 present (Fig. 3B; grey vs black bars); consistent with the known effect of the Arctic mutation to enhance Aβ aggregation (Nilsberth et al., 2001; Philipson et al., 2008) and previous reports of Aβ42 distribution in *Drosophila* models (Finelli et al., 2004; Iijima et al., 2004). DR also had no significant effect on this pattern of Aβ aggregation (Fig. 3B; 1.0 vs 2.0 Y food). GAL4 and UAS control flies did not express soluble or insoluble Aβ42 peptides (Fig. 3).

w^1118^elav/+;UAS-WT4Rtau/+ flies expressed detectable levels of tau protein upon eclosion and this level of expression was maintained throughout life (Fig. 4; anti-tau). Tau phosphorylation, as assessed at three independent epitopes using phosphorylation-dependent antibodies [PHF-1 (Ser396/404); AT8 (Ser199/202); pS422 (Ser422)], was also high upon eclosion in these flies and the degree of phosphorylation was unaltered with age. The level and pattern of tau expression and phosphorylation was identical between DR and fully fed conditions at all time-points (Fig. 4; 1.0 vs 2.0 Y). Tau transcript levels were also unaltered by dietary manipulation (Fig. S1B).

Alterations in diet, therefore, do not regulate the accumulation of Aβ42 or tau or the phosphorylation status of tau in these *Drosophila* models of AD. Hence, the protective effects of DR on tau-induced lifespan reduction do not appear to be mediated by alterations in tau expression or phosphorylation.

### 3.3 DR does not alter negative geotaxis

Since DR does not prevent the abnormal accumulation of Aβ42 peptides or tau, and does not appear to alter neuronal-specific lifespan-shortening effects in the fly, we next investigated the effects of diet on neuronal function downstream of these pathologies. As a behavioural measure of neuronal dysfunction in our *Drosophila* models of AD, locomotor activity was assessed using a negative geotaxis (climbing) assay that has been used previously to characterise several fly models of neurodegenerative disease, including Parkinson's disease (Feany and Bender, 2000) and Alzheimer's disease (Mudher et al., 2004; Crowther et al., 2005).

**Fig. 5:**
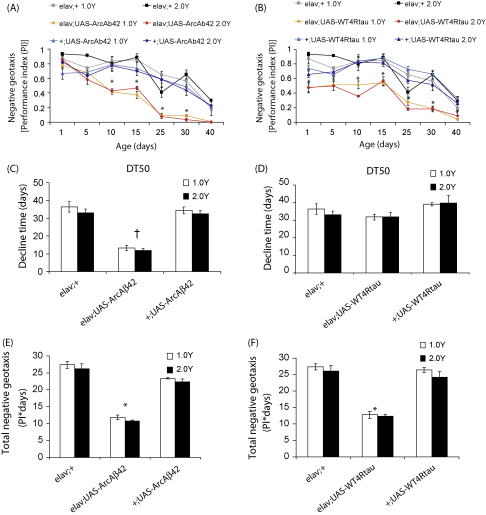
Negative geotaxis behaviour of flies over-expressing Arctic Aβ42 peptides or WT 4R tau on fully fed vs DR SY food. Climbing ability of w^1118^elav/+;UAS-ArcAβ42/+, w^1118^elav/+;UAS-WT4Rtau/+ and control (w^1118^elav/+, w^1118^;UAS-ArcAβ42/+, w^1118^;UAS-WT4Rtau/+) flies on 1.0 vs 2.0 Y medium was assessed across age using several functional descriptors (see Section 2). Data are presented as means ± SEM and were compared using two-way ANOVA and Tukey's honestly significant difference (HSD) post hoc analyses (number of independent tests (*n*) = 3, number of flies per group (*n*_t_) = 43–45). (A and B) Negative geotaxis. Data are expressed as the average performance index (PI) ± SEM for each genotype across age. *P < 0.05 comparing PI of w^1118^elav/+;UAS-ArcAβ42/+ or w^1118^elav/+;UAS-WT4Rtau/+ to that of both GAL4 and UAS control groups at the indicated timepoints (Tukey's HSD). For all genotypes, no significant effect of food on negative geotaxis was observed by two-way ANOVA (*P* = 0.491). (C and D) Decline time (DT). DT50 (time for function to decline to 50% of peak value) constants were interpolated from second-order polynomial curve fits, using the data presented in (A) and (B), for each group. Data are presented as DT ± SEM. ^†^*P* < 0.05 comparing DT50 of w^1118^elav/+;UAS-ArcAβ42/+ to that of both control groups (Tukey's HSD). For all genotypes, no significant difference was observed comparing DT50 values on 1.0 vs 2.0 Y medium (*P* = 0.8971, two-way ANOVA). (E and F) Total negative geotaxis was determined as the area under the curve for each group presented in (A) and (B). Data are shown as total function ± SEM. *P < 0.05 comparing w^1118^elav/+;UAS-ArcAβ42/+ or w^1118^elav/+;UAS-WT4Rtau/+ to that of GAL4 and UAS control groups (Tukey's HSD). For all genotypes no significant difference in total function was observed comparing flies maintained on 1.0 vs 2.0 Y medium (*P* = 0.4692, two-way ANOVA). w^1118^elav/+ flies were the same for both Aβ42 and tau experiments which were run in parallel.

*Drosophila* display an aging-related decline in climbing (Martin and Grotewiel, 2006) and this was apparent in the control flies used in the current study (Fig. 5). Arctic mutant Aβ42 over-expressing flies, however, displayed reduced negative geotaxis (Fig. 5A) and an earlier onset of decline (Fig. 5C; DT50) and total function across age (Fig. 5E) compared to both GAL4 and UAS control flies. Similarly, tau over-expressing flies showed a reduced negative geotaxis (Fig. 5B) and total function across age (Fig. 5F), but the time to decline (Fig. 5D) was not significantly different compared to control flies. Unlike the 4R tau effects on lifespan, however, this tau-induced impairment of climbing ability is a neuronal-specific phenotype, since w^1118^elav/+;UAS-WT4Rtau/+ flies displayed reduced negative geotaxis compared to both the UAS and GAL4 controls.

DR had no significant effect on the impairment of negative geotaxis. A similar negative geotaxis (Fig. 5 A and B), decline time (Fig. 5C and D) and total function (Fig. 5E and F) was observed on 1.0 versus 2.0 Y medium for all genotypes. Hence, dietary manipulation does not alter either the neuronal-specific impairment of negative geotaxis caused by the accumulation of toxic Aβ42 peptides or phosphorylated tau protein, or the aging-related decline in negative geotaxis observed in control flies. Our data, therefore, agree with previously published studies demonstrating dissociation between the effects of DR on lifespan and functional senescence in the fly (Bhandari et al., 2007; Burger et al., 2007), and show further that these observations may also extend to aging-related disease processes.

### 3.4 DR does not prevent neuronal dysfunction in flies over-expressing Arctic Aβ42 or tau protein

Previously published studies have used behavioural defects, such as reductions in negative geotaxis and olfactory conditioning, as indicative measures of neuronal dysfunction in *Drosophila* models of AD, and end-stage measures of neuronal death, such as neuronal loss and apoptosis, to directly characterise neuronal abnormalities (Wittmann et al., 2001; Iijima et al., 2004; Crowther et al., 2005). Direct measures of neuronal activity in these flies have not been reported, however, and may better facilitate the analysis of early stages of AD-related neuronal toxicity in *Drosophila*. Hence, we examined the effects of expression of Arctic Aβ42 or tau proteins and of diet on electrophysiological responses of the nervous system in adult flies.

**Fig. 6:**
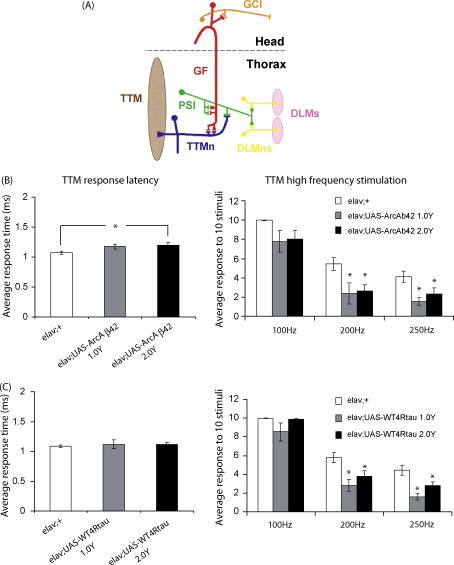
Neuronal electrophysiology of flies over-expressing Arctic Aβ42 peptides or WT 4R tau on fully fed vs DR SY food. (A) A schematic illustration of the *Drosophila* giant fibre system (GFS (Allen et al., 2006) reproduced with permission from Elsevier). Giant fibres (GF; red) relay signals from the brain to the thoracic musculature via mixed electrochemical synapses with the motorneurons (TTMn, blue) of the tergotrancheral muscle (TTM; left), and the peripherally synapsing interneuron (PSI; green) which subsequently forms chemical synapses with the motorneurons (DLMn; yellow) of the dorsal longitudinal muscles (DLM; right). Note only one of the TTMn axons is shown exiting the central nervous system and contacting the muscle on the left hand side and one set of the DLMns and corresponding neuromuscular junctions are depicted on the right hand side. GFS activity was measured, in flies over-expressing (B) Aβ42 peptides or (C) tau protein on fully fed and DR dietary conditions compared to GAL4 controls, by stimulating the giant fibres via electrodes inserted inside the compound eye and recording post-synaptic potentials in the TTM; parameters measured were the latencies from GF stimulation to muscle response (response latency TTM) and the stability of the response to high frequency stimulation at 100, 200 and 250 Hz (high frequency stimulation TTM). Data are presented as means ± SEM. Analysis was performed by one-way ANOVA and Tukey's HSD post hoc comparisons on log-derived data (n = 6-9 for all groups). *P < 0.05 comparing responses of w^1118^elav/+;UAS-ArcAβ42/+ or w^1118^elav/+;UAS-WT4Rtau/+ flies on 1.0 or 2.0 Y medium to that of elav GAL4 controls (Tukey's HSD). No significant differences were observed comparing either w^1118^elav/+;UAS-ArcAβ42/+ or w^1118^elav/+;UAS-WT4Rtau/+ flies on 1.0 Y to those on 2.0 Y medium. Data were not normally distributed at 100 Hz stimulation, but analysis using the non-parametric Kruskal–Wallis ANOVA revealed no significant differences.

The giant fibre system (GFS), which mediates jump-and-flight escape responses, is a useful system for measuring neuronal function, due to its well-defined, easily accessible central nervous system circuitry (Allen et al., 2006). It has been used previously to characterise neuronal dysfunction in fly models of neurodegenerative diseases, such as amyotropic lateral sclerosis (ALS) (Watson et al., 2008). Giant fibres (GF) are interneurons that relay signals from the brain to two main electrochemical synapses in the thoracic ganglion of the fly; one to the motorneuron (TTMn) of the tergotrochanteral muscle (TTM) and the other to the motorneurons (DLMns) of the dorsal longitudinal flight muscle (DLM) via the peripherally synapsing interneuron (PSI) [Fig. 6A (Allen et al., 2006)]. GFS activity was determined in our study by stimulating the giant fibres via electrodes inserted inside the compound eye and recording post-synaptic potentials in the TTM and DLM; parameters measured were the latencies from GF stimulation to muscle response and the stability of the response to high frequency stimulation.

We measured GFS activity in flies over-expressing Arctic Aβ42 or tau proteins on 1.0 and 2.0 Y food at 7 days old, an age at which the deficiency in climbing ability was mild (Fig. 5A and B) and therefore neuronal dysfunction more likely to be modifiable by dietary manipulation. Expression of Arctic Aβ42 peptides significantly increased the response latency measured in the TTM, and inhibited the stability of the TTM response to high frequency stimulation (at 200 and 250 Hz) in comparison to elav GAL4 controls, indicating a defective response of the TTM to GF stimulation (Fig. 6B and Fig. S2). In flies over-expressing tau proteins the response latency in the TTM was comparable between w^1118^elav/+;UAS-WT4Rtau/+ and w^1118^elav/+ flies, but the response to high frequency stimulation was reduced at 200 and 250 Hz (Fig. 6C and Fig. S2). This indicates a relatively mild tau-induced dysfunction in the GFS at this time-point, whereby TTM responses exhibit a normal latency to a single stimulus, but synaptic deficits are apparent when the GFS is challenged with stronger high frequency stimuli. DLM response latencies, however, were unaffected by over-expression of Arctic Aβ42 (Fig. S3A) or WT tau (Fig. S3B), suggesting that the neuronal defects observed may be specific to the GF-TTMn synapse. Dietary manipulation did not alter this pattern of neuronal dysfunction, since response latencies and the stability of responses to high frequency stimulation did not differ on 1.0 and 2.0 Y media both for flies over-expressing Arctic Aβ42 (Fig. 6B and Fig. S2) or tau (Fig. 6C and Fig. S2) in the nervous system.

Our results clearly show that diet does not modulate neuronal toxicity downstream of Aβ42 or tau pathologies in the fly, when measured either indirectly by behavioural analysis or directly by testing neuronal activity.

## 4 Discussion

*D. melanogaster* has proven to be a valuable model organism for studying the molecular mechanisms involved both in the aging process (Bishop and Guarente, 2007) and in various neurodegenerative diseases (Bilen and Bonini, 2005). To explore the connections between the aging process and neurodegenerative disease, we examined the effects of dietary restriction, which extends lifespan in wild-type flies, on lifespan and biochemical and neuronal dysfunction in *Drosophila* models of AD.

Our study extended the characterisation of fly AD models by introducing a direct measure of neuronal activity in response to Aβ42 or tau over-expression in the *Drosophila* nervous system. Other studies have analysed defects in axonal transport in *Drosophila* larvae as a direct measure of neuronal function in flies over-expressing APP (Gunawardena and Goldstein, 2001) or tau proteins (Mudher et al., 2004). These defects may represent abnormal neuronal development in addition to any neurodegeneration in response to AD pathology. We, therefore, developed a direct measure of neuronal dysfunction in adult flies over-expressing Aβ42 or tau proteins, by analysing the electrophysiological responses of the giant fibre system. Pan-neuronal over-expression of Aβ42 or tau proteins resulted in reduced responsiveness of the GFS pathway, compared to both elav GAL4 and UAS controls. There are thus specific defects in neuronal function in the motor system of flies over-expressing these proteins. We propose that this more direct measurement of neuronal dysfunction, in combination with previously established behavioural assays, will better facilitate the investigation of the mechanisms regulating early stages of AD pathogenesis.

Dietary restriction extended the lifespan of both flies over-expressing Aβ42 or tau in the nervous system and of controls. DR thus slowed aging in all the fly genotypes in the study. However, the magnitude of the extension of lifespan by DR was similar in the flies over-expressing Aβ42 and their controls, suggesting that slowing the aging process did not ameliorate the reduction in lifespan induced by Aβ42 expression. Consistent with this observation, dietary manipulation did not alter the abnormal accumulation of Aβ42, with equivalent levels of aggregated Aβ42 peptides present under DR and fully fed dietary conditions. On the other hand, tau-induced lifespan-shortening effects, observed both in elav;UAS-WT4Rtau and +;UAS-WT4Rtau flies compared to elav GAL4 controls, were completely reversed by DR. This could suggest that DR ameliorated the negative effect on lifespan of tau over-expression in neurons. However, an alternative and more likely explanation is that DR rescued the effects of leaky over-expression of tau in tissues other than the nervous system, since both the reduction in lifespan and its amelioration by DR were similar in the tau over-expressing flies and the +;UAS-WT4Rtau controls. In support of this interpretation, DR did not alter tau expression or phosphorylation. Neuronal dysfunction downstream of Aβ42 or tau was not prevented by our dietary restriction protocol. We used both direct (electrophysiology) and indirect (negative geotaxis) measures of neuronal function, and although both were compromised by Aβ42 and tau over-expression, DR did not ameliorate the effects.

Our data thus imply that DR protected against proteotoxic phenotypes in non-neuronal tissues (tau-induced lifespan shortening), but not against neuron-specific abnormalities (Aβ42-induced lifespan shortening; Aβ42 or tau-induced climbing and neuronal activity defects) in response to AD pathologies. Consistent with these findings, DR has been shown to protect against Aβ42 toxicity in worms over-expressing the peptide in non-neuronal tissues (Steinkraus et al., 2008). Our study, therefore, highlights the likely importance of studying the nervous system specifically when trying to understand the mechanisms involved in the pathogenesis of AD and other neurodegenerative diseases using *in vivo* models.

An important finding of our study is that the processes regulating mortality and neurodegeneration can be uncoupled, since DR extended lifespan without ameliorating the toxicity associated with these two *Drosophila* models of AD. Consistent with this observation, flies expressing a mutation in the ADAR (Adenosine Deaminase Acting on RNA) gene display severe, progressive neuro-behavioural impairments, but are not short-lived (Palladino et al., 2000). Moreover, although many studies have suggested that dietary restriction may have neuro-protective properties in rodents (Gillette-Guyonnet and Vellas, 2008), various studies have reported no effect (Bellush et al., 1996; Markowska, 1999; Fitting et al., 2008), a positive effect (Pitsikas et al., 1990; Eckles-Smith et al., 2000; Markowska and Savonenko, 2002), or even a negative effect (Yanai et al., 2004) of DR on aging-associated cognitive decline in these models. Several recent studies have also revealed a complex relationship between the effects of dietary restriction on demographic aging and aging-related functional declines in *Drosophila* (Bhandari et al., 2007; Burger et al., 2007). DR has consistently been shown to extend lifespan in flies (Bhandari et al., 2007; Burger et al., 2007; Grandison et al., 2009), but has also been reported to cause age-dependent reductions in stress resistance (Burger et al., 2007) and no effect on negative geotaxis or olfactory behaviour (Bhandari et al., 2007). This has led to the suggestion that the mechanisms underlying DR-mediated effects on lifespan and aging-related declines in function may dissociate. Our results support these findings since we have observed no effect of DR on the aging-related senescence of negative geotaxis in control flies, and DR also did not ameliorate the neuronal-regulated decline in climbing ability caused by the over-expression of Aβ42 or tau proteins in the *Drosophila* nervous system.

Our findings apparently conflict with previously published studies demonstrating that dietary restriction can improve cognitive function (Halagappa et al., 2007; Wu et al., 2008), prevent amyloid accumulation (Patel et al., 2005; Wang et al., 2005; Halagappa et al., 2007) and reduce tau phosphorylation (Halagappa et al., 2007; Wu et al., 2008) in mouse models of AD. This discrepancy could indicate that the mechanisms underlying the effects of DR on aging differ between organisms (Piper and Partridge, 2007). Alternatively, since DR and intermittent fasting (IF) are hypothesised to mediate their neuro-protective effects at distinct stages of the AD pathological process (Halagappa et al., 2007), the difference may be explained by variations in the particular combination of DR protocol and AD process modelled in the mouse and *Drosophila* studies. Most studies examining the effects of DR on AD pathology have been performed using mice which over-express familial AD mutations in human APP (Wang et al., 2005; Halagappa et al., 2007), or mutant forms of tau in combination with APP and/or presenilin-1 to induce hyperphosphorylation (Halagappa et al., 2007; Wu et al., 2008). Hence processes both upstream and downstream of Aβ production and tau hyperphosphorylation can be examined in these models. Intermittent fasting, despite ameliorating cognitive decline, has no effect on Aβ levels or tau phosphorylation in mice, leading to the hypothesis that the neuro-protective effects of IF act downstream of these pathologies in AD (Halagappa et al., 2007). Conversely, DR reduces Aβ levels (Patel et al., 2005; Wang et al., 2005; Halagappa et al., 2007), possibly by increasing activity of the non-amyloidogenic APP cleaving enzyme α-secretase (Qin et al., 2006), prevents tau hyperphosphorylation (Halagappa et al., 2007; Wu et al., 2008), possibly by reducing activity of the tau kinase cyclin dependent kinase-5 (CDK-5) (Wu et al., 2008), and prevents cognitive decline in these models, suggesting that DR exerts a neuro-protective effect upstream of Aβ and tau pathogenesis. The *Drosophila* models used in our study, however, express Aβ42 peptides or tau in the absence of any confounding factors upstream of their production, hyperphosphorylation or accumulation, and so enable a more definitive analysis of the processes occurring directly downstream of these pathologies in AD. As our dietary restriction protocol most resembles the DR regime used in mice, the absence of neuro-protective effects of DR in our fly models may confirm that any protective effects of DR on AD act firmly upstream of the production of Aβ and tau pathologies.

Overall our findings show overwhelmingly that DR-mediated effects on lifespan and neuronal declines in *Drosophila* may dissociate, and demonstrate that this dissociation particularly applies to Alzheimer's disease-related processes following the initiation of Aβ and tau pathologies. Moreover, our study provides strong support for the hypothesis that the neuro-protective effects of dietary restriction regimes act upstream of Aβ and tau pathogenesis in AD.

## Conflict of interest

The authors have no conflicts of interest to disclose.

## Figures and Tables

**Figure:**
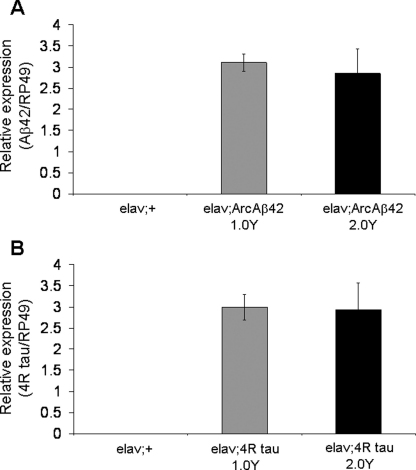


**Figure:**
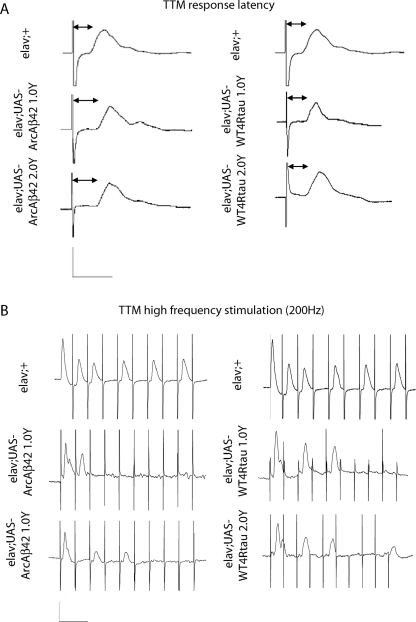


**Figure:**
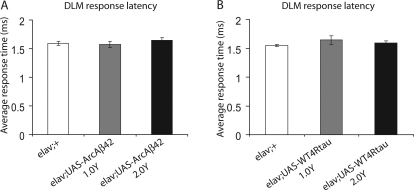


**Table 1:**
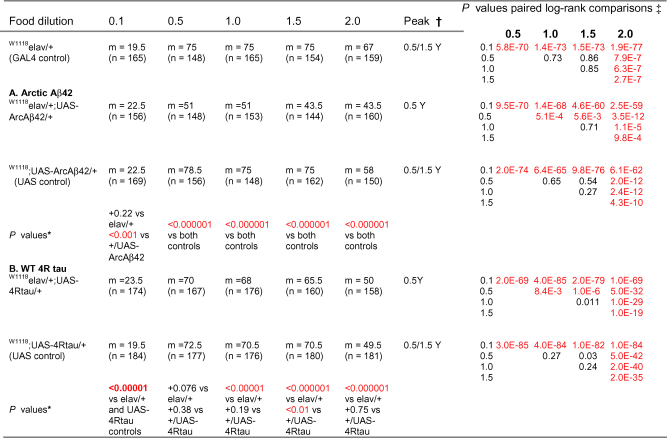
Median lifespan and statistical analysis of Arctic Ab42 and WT 4Rtau overexpressing female flies across a yeast dilution series on SY food.

Survival analysis of Arctic Aβ42 and WT 4R tau over-expressing flies; median lifespan values (*m*), number of flies per group (*n*) and *P* values are indicated. *P values calculated using log-rank tests comparing w^1118^elav/+;UAS-ArcAβ42/+ or w^1118^elav/+;UAS-4Rtau/+ flies with the relevant GAL4 and UAS controls on the same food dilution. Bold *P* values indicate that flies were longer lived than control flies; ^†^Yeast dilution-range at which peak median lifespan was observed for each genotype. ^‡^*P* values of log-rank tests comparing flies across the yeast-dilution series for each genotype, significant values are depicted in red (*P* < 0.01). w^1118^elav/+ flies were the same for both Aβ42 and tau experiments which were run in parallel.
