# Erratum to “Dietary restriction delays aging, but not neuronal dysfunction, in *Drosophila* models of Alzheimer's disease.” [Neurobiol. Aging 32 (2011) 1977–1989]

**DOI:** 10.1016/j.neurobiolaging.2015.05.007

**Published:** 2015-07

**Authors:** F. Kerr, H. Augustin, M.D.W. Piper, C. Gandy, M.J. Allen, S. Lovestone, L. Partridge

In the above-mentioned article, an error in the figure on page 1984 has been noted. In Figure 4 A, the image for the AT8 western blot has been copied to the PHF-1 panel in error. The correct image for the PHF-1 western has now been inserted and can be viewed in the amended figure here:

**Fig. 4:**
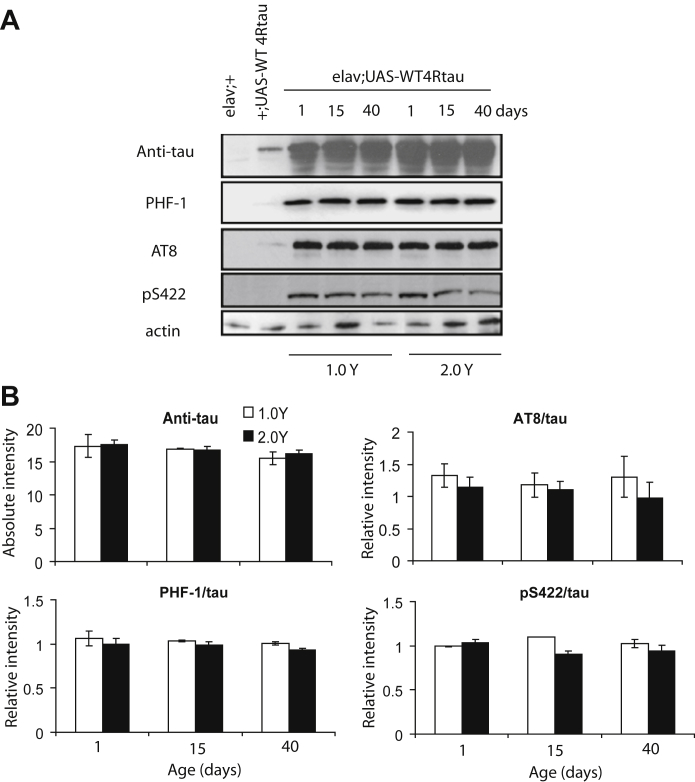
Analysis of fully fed vs DR food effects on tau levels and phosphorylation in flies over-expressing WT human tau. (A) Tau expression and phosphorylation levels were measured by western blotting in control flies (w^1118^elav/+, w^1118^;UAS-4Rtau/+), and at the indicated time points in w^1118^elav/+;UAS-4Rtau/+flies treated on1.0 vs 2.0 Y medium. Primary antibodies were as follows: Anti-tau (total tau; Dako, UK), PHF-1 (phospho-Ser396/404 tau), AT8 (phospho-Ser199/202 tau), pS422(phospho-Ser422 tau) and anti-actin. (B) Phospho-tau levels, in w^1118^elav/+;UAS-4R tau/+flies, were normalised to total tau protein for each sample, and are expressed as average relative intensities ± SEM. Dietary manipulation did not alter the level or pattern of tau phosphorylation across age at Ser396/404 (*P* = 0.412), Ser199/202 (*P* = 0.838) or Ser422 (*P* = 0.677) epitopes (two-way ANOVA).

