# Supplementary material for: Dietary restriction delays aging, but not neuronal dysfunction, in Drosophila models of Alzheimer's disease
Source: Neurobiol Aging. 2011 Nov;32(11):1977–89. doi: 10.1016/j.neurobiolaging.2009.10.015 (PMC3176895; doi:10.1016/j.neurobiolaging.2009.10.015)
Supplement: Supplementary file 1 [file mmc1.doc]

**Supplementary Material**

**Methods**

**Quantitative RT-PCR**

Total RNA from 20 frozen fly heads was extracted using TRIzol (GIBCO) according to the manufacturers’ instructions. Total RNA was then subjected to DNA digestion using DNAse I (Ambion), and mRNA immediately reverse transcribed by using oligo(dT) primer and the Superscript II system (Invitrogen). Samples incubated in the absence of Superscript II were used to control for amplification of residual genomic DNA contamination. Quantitative PCR was performed by using the 7900 Fast Real-Time PCR system and Fast SYBR Green Master Mix (Applied Biosystems), following the manufacturers’ instructions. Four independent samples were generated per genotype and samples were analysed in triplicate with both target gene (Aβ peptide or 4R tau) and control gene (RP49) primers in parallel. Primers used were as follows: Aβ forward, 5’-GATCCTTCTCCTGCTAACC-3’ (5’ end of the signal secretion peptide sequence), Aβ reverse, 5’-CACCATCAAGCCAATAATCG-3’ (3’ end of the Aβ coding sequence), 4R tau forward, 5;-GAGGCGGGAAGGTGCAGATAA-3’ (overlapping the exon 9-exon 10 boundary), 4R tau reverse, 5’-GGATGTTGCCTAATGAGCCAC-3’ (in exon 11), RP49 forward, 5’-ATGACCATCCGCCCAGCATCAGG-3’ and RP49 reverse 5’-ATCTCGCCGCAGTAAACG-3’. Relative quantities of Aβ42 and tau transcripts were determined (relative standard curve method; Applied Biosystems procedures) and normalized to RP49.

**Figure Legends**

Figure S1

Analysis of dietary effects on Aβ42 and tau transcript levels. mRNA was measured by quantitative RT-PCR in (A) ^w1118^elav;UAS-ArcAβ42 or (B) ^w1118^elav;UAS-4Rtau flies on 1.0 and 2.0 Y medium at 7 days old versus their GAL4 controls. Relative Aβ42 or tau expression levels were normalised to RP49 (see supplementary methods). Data are presented as the mean ± SEM and were analysed using ANOVA (n=4). Dietary manipulation had no effect on Aβ42 mRNA levels (*P*=0.71) or tau mRNA levels (*P*=0.9).

Figure S2

Neuronal electrophysiology of flies over-expressing Arctic Aβ42 peptides or WT 4R tau on fully-fed vs DR SY food. Representative traces for (A) TTM response latencies and (B) TTM responses to high frequency stimulation (200 Hz) measured in w^1118^elav/+;UAS-ArcAβ42/+ and w^1118^elav/+;UAS-WT4Rtau/+ flies, fed with 1.0 or 2.0 Y medium, and ^w1118^elav GAL4/+ controls. TTM response latency was increased in Arctic Aβ42 over-expressing flies, but not tau over-expressing flies, compared to elav GAL4 controls (marked with arrows). Vertical scale bars, 50 mV for response latencies, 20 mV for following at 200Hz; horizontal, 2 ms for response latencies, 10ms for following at 200Hz.

Figure S3

DR effects on DLM electrophysiology in *Drosophila* models of AD. GFS activity was measured, in flies over-expressing (A) Aβ42 peptides or (B) tau protein on fully-fed and DR dietary conditions compared to GAL4 controls, by stimulating the giant fibres via electrodes inserted inside the compound eye and recording post-synaptic potentials in the DLM; parameter measured was the latencies from GF stimulation to muscle response (response latency DLM). Data are presented as means ± SEM and were analysed using the non-parametric Kruskal-Wallis one-way ANOVA. No significant differences were observed between groups.
